# Intraperitoneal Fat through GRP78: A Risk Factor for Endometrial Cancer

**DOI:** 10.1155/2016/3496538

**Published:** 2016-10-16

**Authors:** Răzvan Ciortea, Costin Berceanu, Andrei Mihai Măluţan, Radu Mocan, Cristian Iuhas, Carmen Elena Bucuri, Maria Patricia Rada, Dan Mihu

**Affiliations:** ^1^2nd Department of Obstetrics and Gynecology, “Iuliu Hațieganu” University of Medicine and Pharmacy, Cluj-Napoca, Romania; ^2^Department of Obstetrics and Gynecology, University of Medicine and Pharmacy of Craiova, Craiova, Romania

## Abstract

*Introduction. *The identification of biological markers that indicate an increased risk for the development or recurrence of endometrial cancer (EC) in obese women might be useful for decreasing EC mortality and morbidity. Glucose-regulated protein 78 (GRP78) is a major protein of the endoplasmic reticulum expressed in all normal cells. Overexpression of GRP78 has been reported to be a tumoral biomarker. Increased detection of GRP78 is positively correlated with the tumoral stage and prognosis. This study aimed to identify a correlation between intraperitoneal fat, plasma GRP78 levels, and EC.* Materials and Methods.* Two groups of patients were included in the study: group I, 44 patients diagnosed with EC, and group II, 44 patients without gynecological pathology or inflammatory disorders. Visceral fat was determined by ultrasound and plasma GRP78 levels were measured.* Results*. Plasma GRP78 levels were significantly higher in patients with EC compared to the control group. Intraperitoneal fat was in a positive linear correlation with the plasma GRP78 level (*p* < 0.0001).* Conclusion. *The measurement of the GRP78 level associated with the determination of intraperitoneal fat can be a useful predictor for EC.

## 1. Introduction

EC is one of the malignant processes whose etiology begins to be deciphered and understood. The most accepted hypothesis is that of a constant exposure to the action of endogenous and/or exogenous estrogens, an action that is not balanced by progesterone. Obesity is associated with both premenopausal and postmenopausal changes in serum steroid hormone levels.

Visceral adipose tissue increases estrogen concentration through the conversion of androgens to estrogens by aromatase enzyme [1]. The adipocyte is the central element, which integrates multiple metabolic and endocrine signals. This cell is the source for a multitude of cytokines, which play a key role in endometrial carcinogenesis [2]. The identification of biological markers that indicate an increased risk for the development or recurrence of EC in obese women might be useful for decreasing EC mortality and morbidity.

GRP78 is a major protein of the endoplasmic reticulum (ER), which is expressed in normal cells. In adipocytes, GRP78 is a marker for ER stress [3]. GRP78 is a central regulator of ER stress due to its major antiapoptotic role, as well as its ability to control the activation of ER transmembrane stress sensors (IRE1, Perk, and ATF6) [4, 5]. Recent studies have demonstrated that GRP78 plays an important role in tumor development, progression, and chemoresistance [6].

Some cytokines are involved in the pathogenesis of endometrial cancer due to the development of ER stress with the secondary release of GRP78 [7].

GRP78 also regulates intracellular calcium and supports cell survival by an immediate response to insults, having antiapoptotic properties [8]. The reduction of apoptosis allows the affected cells to survive and accumulate additional mutations.

Despite the strong association between obesity and endometrial cancer, it is not clear whether ER stress occurs in the adipocytes of these patients, with this idea underlying the latest studies regarding the elaboration of a new noninvasive method for the diagnosis of EC. The results of these studies have demonstrated that GRP78 levels in visceral adipocytes are correlated with the stage of the disease and patient survival and might be clinically useful as a predictor for EC [9].

## 2. Materials and Methods

The type of the study is case-control and includes 2 groups of patients: group I, 44 patients diagnosed with EC, and group II, 44 patients without gynecological pathology or inflammatory disorders (control group). Patients were enrolled in the study following endometrial biopsy that confirmed EC diagnosis.

Subjects included in the study were assessed for anthropometric data (body mass index (BMI) and abdominal circumference (AC)) and ultrasound examination (US) was also performed in order to determine intraperitoneal fat.

**Figure 1:**
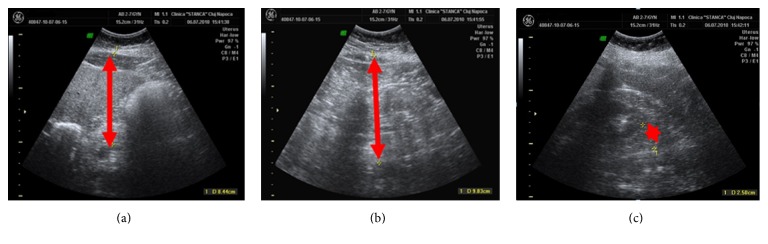
(a) Distance between the inner side of the right abdominal muscle and the splenic vein. (b) Distance between the inner side of the right abdominal muscle and the posterior wall of the aorta. (c) Fat thickness in the posterior wall of the right kidney.

US exploration was performed relating to a method that has been validated by Hirooka et al. [10]. Performed measurements were all introduced in the following formula by which visceral fat area was obtained: 9.008 + 1.191 × [distance between the inner side of the right abdominal muscle and the splenic vein (mm)] + 0.987 × [distance between the inner side of the right abdominal muscle and the posterior wall of the aorta (mm)] + 3.644 × [fat thickness in the posterior wall of the right kidney (mm)] ([10], Figure 1).

Plasmatic GRP78 levels for every subject included in the study were determined on an empty stomach, by drawing 6 mL of venous blood. After obtaining the samples, the serum was centrifuged and stored in 600 *μ*L tubes at a temperature of −60°C until working the probes. Plasmatic levels of GRP78 were determined by the sandwich ELISA technique using the Human GRP78 Immunoassay MBS031039 kit, R&D Systems, USA. The detection rate of this kit was 0.1 *μ*g/mL. No significant cross reactivity or interference between human GRP78 and analogues was found.

Informed consent was obtained from all subjects included in the study. “Iuliu Hațieganu” University of Medicine and Pharmacy ethics committee approved the study.

Normal distribution was tested with the Kolmogorov-Smirnov test. Normally distributed variables are presented as mean ± standard deviation; nonnormally distributed variables are presented as median (interquartile range). For comparison of two means, the *t*-test or Mann–Whitney test was used. For analysis of the relationship between two variables, the Pearson or Spearman correlation coefficient (*r*) was used. Statistical analysis was performed with SPSS 15.0.

## 3. Results

The characteristics of the patients included in the study are described in Table 1.

According to Table 1, patients with EC were older and had an early menarche and late menopause. These patients also had a statistically significantly higher abdominal circumference and increased intraperitoneal fat (*p* < 0.0001).

Plasma GRP78 levels were found to be significantly higher in patients with endometrial cancer compared with nondiseased patients.

We further tested the correlations between GRP78 levels and the main characteristics of the patients. GRP78 values were inversely correlated with age at menarche and directly correlated with the patients' age, onset of menopause, AC, and intraperitoneal fat. In order to avoid confounders, we performed age-controlled correlations, which maintained a similar trend. GRP78 was positively correlated with menopause (*r* = 0.47, *p* < 0.0001), abdominal circumference (*r* = 0.23, *p* = 0.02), and intraperitoneal fat (*r* = 0.38, *p* < 0.0001) (Table 2).

**Figure 2:**
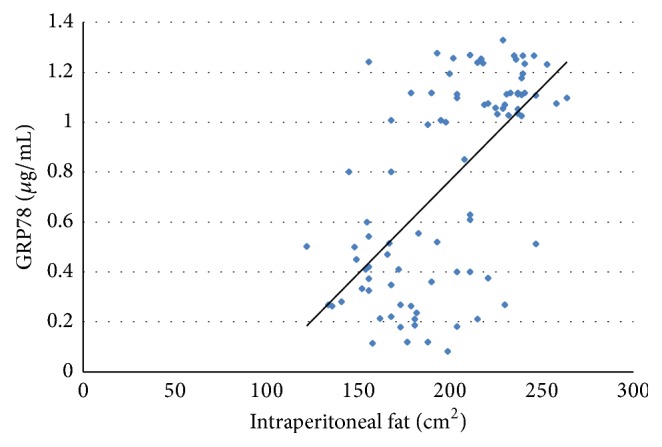
Correlation between GRP78 and intraperitoneal fat.

The positive linear correlation between intraperitoneal fat and plasma GRP78 levels can be seen in Figure 2.

In order to establish the influence of the other variables on* GRP78*, stepwise multivariate linear regression analysis was performed. The following variables were entered in regression: age, menopause, abdominal circumference, and intraperitoneal fat (Table 3).

According to Table 3, age, menopause, and intraperitoneal fat proved to be independent predictors of high serum GRP78 levels, directly influencing their variability. Considering the three parameters together, old age, age at menopause, and increased intraperitoneal fat influence 65% (*R* ^2^-adjusted = 0.65) of GRP78 variance.

## 4. Discussions

EC is a very frequent pathological entity that has an ascending incidence particularly in the high living standard countries [1].

Regarding epidemiological evidence, lifestyle characterized by low physical activity and obesity is a factor that plays a well-established role in EC etiology.

In individuals with morbid obesity, adipose tissue is an important trigger that induces adipokine synthesis. Among most frequent secretion products of the adipocyte, PGE2, IL8, and IL6 presented a higher plasmatic level [11]. There also was a positive correlation between BMI and IL8 levels [12].

Even though increased visceral fat represents a well-defined risk factor for cardiac and metabolic diseases, its role in the etiopathogeny of EC is insufficient quantified [13]. It is not clearly defined why disposition of the adipose tissue particularly in abdominal region generates increased risk of the abovementioned pathologies. Standardized increase in BMI is directly proportional with rising of EC risk [14, 15].

Visceral fat as a prognostic marker of EC progression is an unexplored area. During cellular perturbations, such as those encountered by adipocytes in obesity [16], the protein load in the ER exceeds its folding capacity, resulting in the retention of misfolded proteins within the ER and, consequently, in ER stress. Activation of the unfolded protein response (UPR) alleviates ER stress by decreasing general protein translation and increasing the protein folding capacity of the ER [17].

Recent studies described contradictory results regarding estradiol influence on stress ER. Plasmatic level of GRP78 secreted from stress ER remains unmodified after estradiol addition to Ishikawa cells [18]. On the other hand, GRP78 levels in murine uteri have been shown to be upregulated by estrogen and estrogen-like compounds. However, these effects seen in mice appear to be independent of canonical estrogen-receptor mediated pathways [19].

This study supports a correlation between visceral fat, plasmatic GRP78 levels, and EC, with the central element of this association being the adipocyte whose implication in the EC pathogenesis is generally accepted [20, 21].

There were described mechanisms through which the adipocyte influences the metastatic capacity of ovarian tumoral cells, consequently to lipolytic modifications [22]. Overweight induces GRP78 activation in subcutaneous adipocytes in oncogenic and nononcogenic human models [23].

The association between visceral obesity and EC can be due to ER stress in adipocytes. ER stress is an adaptive reaction that normally occurs in any cell required to process more molecules than usual in a time unit. In this situation, there is the risk that some incompletely processed molecules may generate an adaptive reaction in the ER, consisting of conformational defects in molecules, which might prevent traffic through this structure [24].

Given the critical role that GRP78 plays in tumor development and progression, it can be speculated that ER stress and GRP78 in adipocytes may elicit increased adipokine production that acts on adjacent tumor cells and/or the local microenvironment and enhances progression. Conversely, EC cells experiencing ER stress may undergo changes in expression of factors that impacts their local environment, giving rise to ER stress in neighboring cells including visceral adipocytes.

GRP78 is involved in cell survival having antiapoptotic properties [25] generating mutations. Plasma estrogen levels, which are directly correlated with plasma GRP78 levels, suggest the implication of GRP78 in the pathogenesis of EC [26].

This study supports the idea that intraabdominal fat is directly correlated with plasma GRP78 levels, as well as with EC, suggesting the implication of GRP78 in the pathogenesis of EC.

By quantifying the role of the secretion products of the adipocyte in the EC pathogenesis, new predictive factors could be proposed for this pathological entity. Given that a reliable screening test for EC was not yet described, in obese patients, assessment of visceral fat in correlation with GRP78 could narrow the group of patients with EC risk.

## Figures and Tables

**Table 1:** Characteristics of the patients included in the study.

Characteristic	Group	*N*	Mean	*p* value	Standard deviation	95% confidence interval for the mean		Minimum	Maximum
						Lower bound	Upper bound		
Age	0	44	55.11	<0.0001	8.47	52.54	57.69	39	70
	1	44	71.84		5.69	70.11	73.57	39	83
	Total	88	63.48		11.05	61.14	65.82	39	83

Menarche	0	44	13.02	0.002	1.09	12.69	13.35	11	15
	1	44	12.25		1.16	11.90	12.60	10	15
	Total	88	12.64		1.19	12.39	12.89	10	15

Menopause	0	44	46.00	Ns	3.03	45.08	46.92	40	54
	1	44	51.95		3.38	50.93	52.98	40	59
	Total	88	48.98		4.38	48.05	49.90	40	59

BMI	0	44	31.23	Ns	2.88	30.35	32.10	23	37
	1	44	31.89		2.93	31.00	32.78	26	38
	Total	88	31.56		2.91	30.94	32.17	23	38

AC (cm)	0	44	90.86	<0.0001	12.75	86.99	94.74	70	118
	1	44	104.05		11.30	100.61	107.48	86	124
	Total	88	97.45		13.69	94.55	100.36	70	124

Intraperitoneal fat (cm^2^)	0	44	176.14	<0.0001	4.18	167.70	184.57	122	247
	1	44	222.25		3.59	215.01	229.49	156	264
	Total	88	199.19		3.69	191.86	206.53	122	264

GRP78 (*µ*g/mL)	0	44	0.379	<0.0001	0.188	0.322	0.436	0.081	0.85
	1	44	1.138		0.095	1.110	1.167	0.989	1.329
	Total	88	0.759		0.410	0.672	0.846	0.081	1.329

Case: 1; Control: 0; AC: abdominal circumference.

**Table 2:** Correlations between GRP78 and patient characteristics.

	GRP78 age	
	Uncontrolled correlations	Controlled correlations
*Age*		
*r*	0.73	
*p*	<0.0001	
*Menarche*		
*r*	−0.30	−0.17
*p*	0.005	0.11
*Menopause*		
*r*	0.63	0.47
*p*	<0.0001	<0.0001
*G (kg)*		
*r*	0.07	0.01
*p*	0.51	0.89
*BMI*		
*r*	0.10	−0.02
*p*	0.33	0.84
*AC (cm)*		
*r*	0.42	0.23
*p*	<0.0001	0.02
*Intraperitoneal fat (cm* ^*2*^)		
*r*	0.63	0.38
*p*	<0.0001	<0.0001

*r*: Pearson correlation coefficient.

**Table 3:** Multiple linear regression analysis of parameters associated with GRP78 levels (dependent variable: GRP78).

Independent variables	Coefficient	Standard error	*t*	*p* value
(Constant)	−2.29			
Age	0.01	0.002	5.593	<0.0001
Menopause	0.02	0.007	4.060	0.0001
AC (cm)	0.000003	0.002	0.00166	0.9987
Intraperitoneal fat (cm^2^)	0.002	0.0009	2.908	0.0047
Coefficient of determination *R* ^2^	0.67			
*R* ^2^-adjusted	0.65			

*p* < 0.0001.

## References

[1] Greenberg A. S., Obin M. S. (2006). Obesity and the role of adipose tissue in inflammation and metabolism. *The American Journal of Clinical Nutrition*.

[2] Modugno F., Ness R. B., Chen C., Weiss N. S. (2005). Inflammation and endometrial cancer: a hypothesis. *Cancer Epidemiology Biomarkers & Prevention*.

[3] Gonzalez-Gronow M., Selim M. A., Papalas J., Pizzo S. V. (2009). GRP78: a multifunctional receptor on the cell surface. *Antioxidants and Redox Signaling*.

[4] Wu J., Kaufman R. J. (2006). From acute ER stress to physiological roles of the unfolded protein response. *Cell Death and Differentiation*.

[5] Ni M., Lee A. S. (2007). ER chaperones in mammalian development and human diseases. *FEBS Letters*.

[6] Teske B. F., Wek S. A., Bunpo P. (2011). The eIF2 kinase PERK and the integrated stress response facilitate activation of ATF6 during endoplasmic reticulum stress. *Molecular Biology of the Cell*.

[7] Dal Maso L., Augustin L. S. A., Karalis A. (2004). Circulating adiponectin and endometrial cancer risk. *The Journal of Clinical Endocrinology & Metabolism*.

[8] Pootrakul L., Datar R. H., Shi S.-R. (2006). Expression of stress response protein Grp78 is associated with the development of castration-resistant prostate cancer. *Clinical Cancer Research*.

[9] Matsuo K., Gray M. J., Yang D. Y. (2013). The endoplasmic reticulum stress marker, glucose-regulated protein-78 (GRP78) in visceral adipocytes predicts endometrial cancer progression and patient survival. *Gynecologic Oncology*.

[10] Hirooka M., Kumagi T., Kurose K. (2005). A technique for the measurement of visceral fat by ultrasonography: comparison of measurements by ultrasonography and computed tomography. *Internal Medicine*.

[11] Fain J. N., Madan A. K., Hiler M. L., Cheema P., Bahouth S. W. (2004). Comparison of the release of adipokines by adipose tissue, adipose tissue matrix, and adipocytes from visceral and subcutaneous abdominal adipose tissues of obese humans. *Endocrinology*.

[12] Fortuño A., Rodríguez A., Gómez-Ambrosi J., Frühbeck G., Díez J. (2003). Adipose tissue as an endocrine organ: role of leptin and adiponectin in the pathogenesis of cardiovascular diseases. *Journal of Physiology and Biochemistry*.

[13] Schmandt R. E., Iglesias D. A., Co N. N., Lu K. H. (2011). Understanding obesity and endometrial cancer risk: opportunities for prevention. *American Journal of Obstetrics and Gynecology*.

[14] Münstedt K., Wagner M., Kullmer U., Hackethal A., Franke F. E. (2008). Influence of body mass index on prognosis in gynecological malignancies. *Cancer Causes and Control*.

[15] Temkin S. M., Pezzullo J. C., Hellmann M., Lee Y.-C., Abulafia O. (2007). Is body mass index an independent risk factor of survival among patients with endometrial cancer?. *American Journal of Clinical Oncology: Cancer Clinical Trials*.

[16] Özcan U., Cao Q., Yilmaz E. (2004). Endoplasmic reticulum stress links obesity, insulin action, and type 2 diabetes. *Science*.

[17] Pfaffenbach K. T., Lee A. S. (2011). The critical role of GRP78 in physiologic and pathologic stress. *Current Opinion in Cell Biology*.

[18] Guzel E., Basar M., Ocak N., Arici A., Kayisli U. A. (2011). Bidirectional interaction between unfolded-protein-response key protein HSPA5 and estrogen signaling in human endometrium. *Biology of Reproduction*.

[19] Ray S., Hou X., Zhou H.-E., Wang H., Das S. K. (2006). Bip is a molecular link between the phase I and phase II estrogenic responses in uterus. *Molecular Endocrinology*.

[20] Hotamisligil G. S. (2010). Endoplasmic reticulum stress and the inflammatory basis of metabolic disease. *Cell*.

[21] Ciortea R., Angheluta L. M., Pintican D. (2014). Glucose-regulated protein 78, an important biomarker in cancer etiopathogenesis. *Gineco.eu*.

[22] Nieman K. M., Kenny H. A., Penicka C. V. (2011). Adipocytes promote ovarian cancer metastasis and provide energy for rapid tumor growth. *Nature Medicine*.

[23] Sharma N. K., Das S. K., Mondal A. K. (2008). Endoplasmic reticulum stress markers are associated with obesity in nondiabetic subjects. *The Journal of Clinical Endocrinology & Metabolism*.

[24] Koong A. C., Chauhan V., Romero-Ramirez L. (2006). Targeting XBP-1 as a novel anti-cancer strategy. *Cancer Biology and Therapy*.

[25] Pootrakul L., Datar R. H., Shi S.-R. (2006). Expression of stress response protein Grp78 is associated with the development of castration-resistant prostate cancer. *Clinical Cancer Research*.

[26] Ciortea R., Măluţan A. M., Angheluta L. M. (2015). Influence of melatonin on plasma glucose-regulated protein 78 levels in ovariectomized female rats. *Gineco.eu*.

